# Comparative Analysis of CpG Islands in Four Fish Genomes

**DOI:** 10.1155/2008/565631

**Published:** 2008-04-29

**Authors:** Leng Han, Zhongming Zhao

**Affiliations:** ^1^Virginia Institute for Psychiatric and Behavioral Genetics, Virginia Commonwealth University, Richmond, VA 23298, USA; ^2^State Key Laboratory of Genetic Resources and Evolution, Kunming Institute of Zoology, Chinese Academy of Sciences, Kunming, Yunnan 650223, China; ^3^Graduate School, Chinese Academy of Sciences, Beijing 100039, China; ^4^Department of Human Genetics, Virginia Commonwealth University, Richmond, VA 23298, USA; ^5^Center for the Study of Biological Complexity, Virginia Commonwealth University, Richmond, VA 23284, USA

## Abstract

There has been much interest in CpG islands (CGIs), clusters of CpG dinucleotides in GC-rich regions, because they are considered gene markers and involved in gene regulation. To date, there has been no genome-wide analysis of CGIs in the fish genome. We first evaluated the performance of three popular CGI identification algorithms in four fish genomes (tetraodon, stickleback, medaka, and zebrafish). Our results suggest that Takai and Jones' (2002) algorithm is most suitable for comparative analysis of CGIs in the fish genome. Then, we performed a systematic analysis of CGIs in the four fish genomes using Takai and Jones' algorithm, compared to other vertebrate genomes. We found that both the number of CGIs and the CGI density vary greatly among these genomes. Remarkably, each fish genome presents a distinct distribution of CGI density with some genomic factors (e.g., chromosome size and chromosome GC content). These findings are helpful for understanding evolution of fish genomes and the features of fish CGIs.

## 1. Introduction

CGIs are clusters of CpG dinucleotides in GC-rich regions, usually ~1 kb long [1]. They are identified in the promoter regions of approximately 50% of genes in vertebrate genomes and are considered gene markers. CpG islands are usually unmethylated in a genome, especially in the promoter regions [2], in contrast, ~80% of CpG dinucleotides in the mammalian genomes are methylated [2, 3]. The mutation rate of the methylated CpG (5mCpG) to TpG was estimated to be ~10–50-folds higher than that of the unmethylated CpG site due to a high rate of deamination at the 5mCpG, which subsequently leads to an overall loss of CpG dinucleotides and a potential loss of CGIs [4, 5]. Recent studies found that CGIs may be methylated under an abnormal condition or even in normal cells. Weber et al. [6] found an association of DNA methylation in CpG-poor promoters in the germline with an increased loss of CpG dinucleotides, implying that characteristics of the CGIs have been weakened or even vanished in the course of evolution. Methylation of promoter-associated CpG islands has been found to play an important role in gene silencing, genomic imprinting, X-chromosome inactivation, and carcinogenesis [7, 8].

Antequera and Bird [9] hypothesized that CGIs arose at the dawn of vertebrate evolution and gene-associated CGIs might be lost due to de novo methylation. The number of CGIs varies greatly in mammalian genomes, for example, ~20,500 mouse CGIs compared to ~37,500 human CGIs and ~58,300 dog CGIs, even though they have similar gene numbers and genome sizes. Comparisons of CGIs among a few model mammalian genomes, especially between the human and mouse, have been performed [9–11]. Those studies revealed that the mouse has undergone a faster CpG loss than the human, thus, has fewer CGIs and weaker CGI characteristics. The loss of CGIs in those studies was largely attributed to the methylation. However, methylation could not explain all the differences of CGIs in vertebrate genomes. For example, the dog genome has a much larger number of CGIs and higher CGI density than other mammalian genomes, but this large difference is mainly caused by many more CGIs in the dog’s noncoding regions (unpublished data). The number of gene-associated CGIs in the dog genome is not much different from that in other mammalian genomes. Moreover, previous analyses of CGIs in the chicken genome revealed a high concentration of CGIs on microchromosomes [12, 13]. These results suggest that some other genomic factors might have also played important roles in the course of CGI evolution.

Animals evolved in the direction of cold-blooded vertebrates to warm-blooded vertebrates. Bird’s early study [3] found a different CpG distribution among vertebrates and found that the ratio of the observed over the expected CpGs (Obs_CpG_/Exp_CpG_) in cold-blooded vertebrates (e.g., fish) was much higher than in warm-blooded vertebrates (e.g., human and mouse), suggesting a lower or even lack of methylation process in cold-blooded vertebrates. So far, it remains largely unknown of CGIs and their distribution in nonmammalian genomes, especially in the fish, reptile, and amphibian. Fish, which is among the first appeared vertebrates on earth, still has ancient noncoding elements conserved with the human [14]. Several fish genomes have been sequenced recently. This provides us an opportunity to examine and compare CGIs in fish genomes.

In 1987, Gardiner-Garden and Frommer [15] first proposed an algorithm for scanning CGIs in a DNA sequence. This algorithm, which uses three search parameters GC content, Obs_CpG_/Exp_CpG_, and length, has been widely applied in numerous analyses of CGIs in single genes or small sets of genomic sequences. However, this algorithm significantly inflates the number of CGIs because many repeats (e.g., *Alu*), which are abundant in vertebrate genomes, also meet the criteria of this algorithm. To solve this problem, Takai and Jones [7] performed a systematic evaluation of the three parameters in Gardiner-Garden and Frommer’s algorithm and provided an optimal set of parameters. Starting from here, we abbreviated these two algorithms as “GF” and “TJ” to save space. TJ’s algorithm can effectively exclude the false positive results from repeats and also more likely identify CGIs associated with the 5′ end of genes [7]. However, their evaluation was mainly based on the human genome. Whether it is suitable for other genomes, especially the cold-blooded vertebrates, needs further investigation. More recently, Hackenberg et al. [16] developed a new algorithm, namely, CpGcluster, that entirely depends on the statistical significance of a CpG cluster from the random sequence in the same genome. One of its major features is no requirement of minimum length for a CGI. Besides these three major algorithms, there are some other applications such as CpGProD [17] and CpGIE [18], which are essentially based on TJ’s algorithm. These applications give out similar CGI findings because they modified only some of the parameters (e.g., size of the sliding window, number of steps for scanning CGIs).

In this study, we first evaluated the performance of three popular CGI identification algorithms. Then, we performed a systematic analysis of CGIs in five publicly available fish genomes (tetraodon, stickleback, medaka, zebrafish, and fugu) and examined CGI density at the chromosome level in four of them (except for fugu because of the lack of assembled chromosome data). We also compared the features of fish CGIs to other vertebrate genomes.

## 2. Materials and Methods

### 2.1. Genome Sequences and other Genome Information

We downloaded the reference sequences of five fish genomes (tetraodon, stickleback, medaka, zebrafish, and fugu) from UCSC Genome Browser (http://genome.ucsc.edu/). The genomic sequences have been assembled into chromosomes in four fish genomes (tetraodon, stickleback, medaka, and zebrafish) but not in the fugu genome. Therefore, we analyzed and compared CpG islands mainly in the four fish genomes in this study. The number of genes in each fish genome was retrieved from the Ensembl database (http://www.ensembl.org/, build 49).

We used the EMBOSS package [19] to calculate the genome size, GC content, and Obs_CpG_/Exp_CpG_ in these genomes. Table 1 summarizes the statistics of these fish genomes.

### 2.2. Algorithms for the Identification of CGIs

We scanned CGIs in genomic sequences using three algorithms. First, we applied TJ’s algorithm, which is optimized for searching CGIs associated with the 5′ end of genes in the human and other mammalian genomes. Its search criteria are: GC content ≥55%, Obs_CpG_/Exp_CpG_ ≥ 0.65, and length ≥ 500 bp. Second, we used GF’s algorithm: GC content > 50%, Obs_CpG_/Exp_CpG_ > 0.60, and length > 200 bp. These parameters were from the original publication [15], but we applied them to only the nonrepeat portions of the genomes as many repeats in the genomes also meet these parameters [1, 20].

In TJ’s algorithms, there are eight iterative steps to scan all the possible CGIs in a genome. (1) Set a window size to be 200 bases at the start position of a sequence and calculate GC content (%) and Obs_CpG_/Exp_CpG_ in the first window. Here, Obs_CpG_/Exp_CpG_ = N_CpG_/(N_C_ × N_G_) × N where N_CpG_, N_C_, N_G_, and N are, respectively, the number of dinucleotide CpGs, nucleotide Cs, nucleotide Gs, and all nucleotides (A, C, G, and T) in the sequence (i.e., 200 nucleotides). Shift the window 1 base each time until the window meets the criteria for a CGI. (2) Once a seed window (i.e., it meets the criteria) is found, move the window 200 bases afterward and then evaluate the new window again. (3) Repeat step 2 until the window does not meet the criteria. (4) Shift the last window 1 base each time toward the 5′ end until it meets the criteria. (5) Evaluate the whole segment (i.e., from the start position of the seed window to the end position of the current window). If it does not meet the criteria, trim 1 base from each side until it meets the criteria. (6) Connect two individual CGI fragments if they are separated by less than 100 bases. (7) Repeat step 5 to evaluate the new sequence segment until it meets the criteria. (8) Reset start position immediately after the CGI identified at step 7 and go to step 1. This computational procedure has been implemented in the CpG island searching program (CpGi130) [21], which was used in this study. Similar steps were implemented for GF’s algorithm.

Third, we applied CpGcluster developed by Hackenberg et al. [16] to scan CGIs in genomes. There are two main steps in the implementation of CpGcluster. (1) Search CpG clusters based on statistical properties of the physical distances between neighboring CpG dinucleotides on a DNA sequence. (2) Assign a *P*-value, the probability of such a cluster appearing by chance in a random sequence, to each CpG cluster in step 1. Those clusters with a *P*-value less than 10^−5^ were considered statistically significant CGIs. No minimum size length is required in CpGcluster.

## 3. Results

### 3.1. Evaluation of Algorithms on CGI Identifications in Fish Genomes

**Figure 1:**
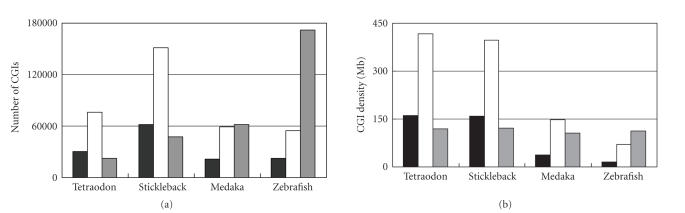
Evaluation of algorithms on CGI identifications in four fish genomes. (a) Number of CGIs, (b) CGI density (per Mb). The black, white, and gray bars represent the CGIs identified by Takai and Jones’ [7], Gardiner-Garden and Frommer’s [15], and CpGcluster [16] algorithms, respectively.

We evaluated whether the three major algorithms could reliably identify CGIs in fish genomes. Figure 1 shows the numbers of CGIs and the CGI densities identified by these algorithms in four fish genomes (tetraodon, stickleback, medaka, and zebrafish). Overall, these three algorithms gave out much different numbers of CGIs and, correspondingly, CGI densities. First, we compared the results from GF’s and TJ’s algorithms. GF’s algorithm gave out a much larger number of CGIs than TJ’s, which is expected because the former one used much less stringent criteria (e.g., minimum length 200 bp). Such a large difference has been shown in other studies [11, 16]. It is important to note that, although the large difference was observed in each genome (e.g., in the tetraodon, 75 771 CGIs by GF’s algorithm versus 30 175 by TJ’s algorithm), both algorithms gave out the same comparative results among genomes. For example, both algorithms had the same rank of CGI density: tetraodon > stickleback > medeka > zebrafish. Because the number of CGIs identified by GF’s algorithm is always substantially greater than the number of genes in a mammalian genome [1, 20], which also holds in these four fish genomes here (Table 1, gene number ranged from 20 159–28 639), we consider TJ’s algorithm is more suitable for CGI identification in fish genomes.

We next compared the performance of TJ’s algorithm with CpGcluster. These two algorithms generated different results too. The number of CGIs in the zebrafish was 171 865 by CpGcluster, which is 7.7 times that (22 392) by TJ’s algorithm. Conversely, we found a smaller number of CGIs in the stickleback (47 386) by CpGcluster than that (61 768) by TJ’s algorithm. Furthermore, CGI density, which was measured by the average counts of CGIs in 1-Mb sequence, was nearly the same among the four genomes by using CpGcluster, opposite to the great variation found by TJ’s algorithm (Figure 1(b)). Because CpGcluster identified a CGI by its statistical significance from a random sequence in the same genome, its CGIs were identified relative to the genome characteristics. This likely eliminated the influence of some genomic factors on CGIs. For example, the GC content of the zebrafish genome (36.5%) is much lower than that (45.9%) of the tetraodon genome. According to the traditional definition, CGIs are in the GC-rich regions. This means that, without considering other factors, it is expected to find more CGIs in tetraodon than in zebrafish. This indeed was observed by both TJ’s and GF’s algorithms. However, CpGcluster evaluated the CpG clusters (i.e., CGIs) from the sequence background in the same genome, which effectively eliminated the difference between the genomes. This is why we observed similar CGI density among the four fish genomes by the CpGcluster.

**Figure 2:**
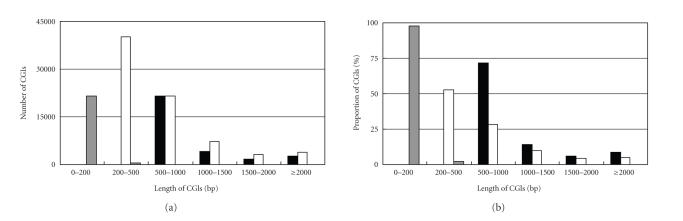
Length distribution of CGIs identified by the three algorithms. The data were based on the tetraodon sequences, but the similar pattern was observed in other fish genomes. The black, white, and gray bars represent the CGIs identified by Takai and Jones’ [7], Gardiner-Garden and Frommer’s [15], and CpGcluster [16] algorithms, respectively.

We further examined the length distribution of CGIs identified by the three algorithms. In the tetraodon, 53% of GF’s CGIs had length between 200 and 500 bp while 47% longer than 500 bp. As expected, TJ’s algorithm had longer CGIs: 72% CGIs whose lengths were between 500–1000 bp and 28% were >1000 bp. Surprisingly, almost all the CGIs identified by CpGcluster (98%) were shorter than 200 bp (Figure 2). The similar length distribution was observed in other fish genomes. It has been widely accepted that CGIs are often longer than 500 bp [1, 2]. Therefore, at least for comparative genomic analysis, we suggest that TJ’s algorithm is more suitable for identification of CGIs in fish genomes than the other two algorithms.

### 3.2. CGIs vary Greatly among Fish Genomes

According to our evaluation above, we applied Takai and Jones’ algorithm to identify CGIs in fish genomes. Table 1 shows the number of CGIs and CGI density in each genome. The number of CGIs, which ranged from 21 522 (medaka) to 61 768 (stickleback), varied greatly among the five fish genomes. Strikingly, there were 61 768 CGIs in only 391 Mb stickleback genomic sequences compared to only 22 392 CGIs in 1524 Mb zebrafish sequences. Because the genome size varied greatly, we calculated the CGI density and made another comparison. Again, CGI density varied greatly: CGI density in both the tetraodon (161.6 CGIs/Mb) and stickleback (157.8 CGIs/Mb) was approximately 11-fold higher than that in the zebrafish (14.7 CGIs/Mb).

**Figure 3:**
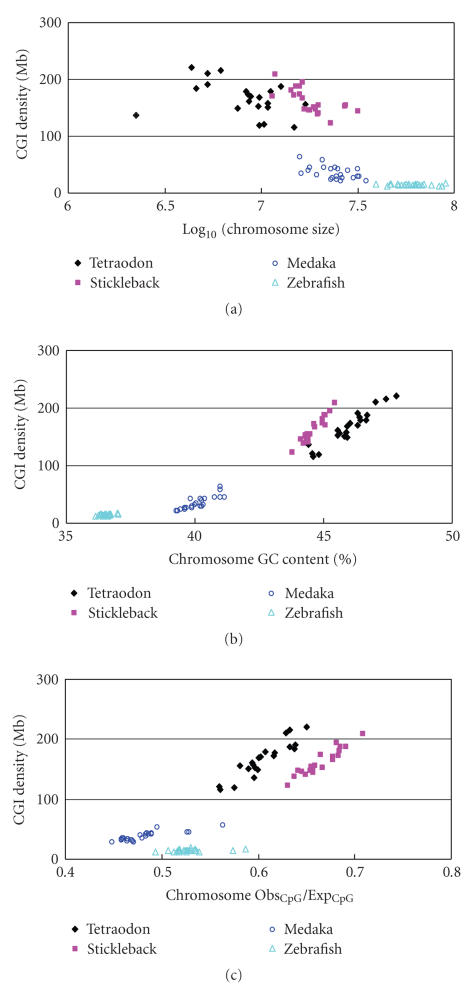
Distinct features of CpG islands in four fish genomes. (a) Distribution of CGI density (per Mb) with Log_10_ (chromosome size), (b) distribution of CGI density (per Mb) with chromosome GC content (%), (c) distribution of CGI density (per Mb) with chromosome Obs_CpG_/Exp_CpG_.

We next examined CGI density at the chromosomal level in the four fish genomes (tetraodon, stickleback, medaka, and zebrafish). The fugu data were excluded because of the lack of assembled chromosomes. Interestingly, when we plotted CGI density over some chromosome parameters (size, GC content, and Obs_CpG_/Exp_CpG_), we found that the chromosomes from each fish genome clustered but overall they were separated from other fish genomes (Figure 3). This distinct pattern is especially obvious in the plots of CGI density over chromosome GC content (Figure 3(b)) and over chromosome Obs_CpG_/Exp_CpG_ (Figure 3(c)). Such a feature was not observed in the mammalian genomes [22]. Moreover, the plots in Figure 3 indicate a significant negative correlation between CGI density and log_10_(chromosome size) (*r* = −0.81, *P* = 5.5 × 10^−23^), a significant positive correlation between CGI density and chromosome GC content (*r* = 0.96, *P* = 7.9 × 10^−50^), and, as expected, a significant positive correlation between CGI density and chromosome Obs_CpG_/Exp_CpG_ (*r* = 0.86, *P* = 2.4 × 10^−28^) in fish genomes. However, the relationship between CGI density and chromosome size in each genome is much different (Figure 3(a)). In both the tetraodon and stickleback, CGI density was high and also varied greatly. Conversely, CGI density in the zebrafish and medaka had a small variation among their chromosomes (Figure 3(a)). Overall, the correlation between CGI density and chromosome GC content was strong regardless only one genome or all four fish genomes being considered (Figure 3(b)), suggesting that chromosome GC content is likely a major genetic factor influencing CGI density.

Among the five fish species we studied, zebrafish first diverged about 110–160 million years ago (MYA) [23] and its genome size is the largest. An interesting feature in the zebrafish is that its CGI density is similar among its chromosomes. This feature might be attributed to its similar chromosome GC content (Figure 3(b)). Three closely related fish species, tetraodon, fugu, and stickleback, diverged 60–80 MYA and are evolutionarily related. They have similar genomic features such as small genome size, high GC content, and high Obs_CpG_/Exp_CpG_ (Table 1). Tetraodon and stickleback had the similar distribution in Figure 3. Medaka was most recently evolved and has intermediate genomic features compared to other four fish species.

## 4. Discussion

In this study, we performed the first systematic survey of CGIs in four fish genomes. We found that the number of CGIs and the CGI density varied greatly in these fish genomes. Moreover, the CGI density in these fish genomes was significantly correlated with some genomic factors at the chromosome level such as chromosome size, GC content, and Obs_CpG_/Exp_CpG_. It seems that the correction between the CGI density and GC content is strong. However, this work is still preliminary. Future work is warranted for identifying other genomic factors that are also correlated with CGI distribution and for evaluating which genomic factor(s) prevailed in the course of CGI evolution in fish genomes.

The extent of CGI variation among fish genomes is stronger than other vertebrate genomes. We did a similar analysis of CGIs in 9 mammals (human, chimpanzee, macaque, mouse, rat, dog, cow, horse, and opossum) whose whole genomes have been assembled. Among the 9 mammalian genomes, the highest CGI density (25.3 CGIs/Mb, the dog genome) was 3.4 times the lowest CGI density (7.5 CGIs/Mb, the opossum genome). This is much weaker than the ~11-fold difference observed in the fish genomes (Table 1). Interestingly, each fish genome had a distinct distribution of CGI density at the chromosome level (Figure 3); a pattern was not found in mammalian genomes. This unique feature might be caused by genetic (sequence composition evolution) and environmental factors such as water temperature, speed of flow, extent of light in different depth of water during the long evolutionary period after the divergence of common ancestor of fishes.

Strong CpG depletion is a common feature in mammalian genomes, for example, ~75–80% of CpG dinucleotides were depleted in the human and mouse genomes [1, 24, 25]. However, CpGs presented much more frequently in fish than in mammalian genomes. The Obs_CpG_/Exp_CpG_ ratio ranged from 0.479 to 0.662 in these fish genomes (Table 1), remarkably higher than that in mammals. Methylation and subsequent deamination is a main process to cause CpG depletion in warm-blooded vertebrate genomes. The Obs_CpG_/Exp_CpG_ ratio in fishes may suggest a similar influence of methylation/deamination in cold-blooded vertebrates, but the extent was much weaker. A further comparative genomics analysis including gene information may help us uncover how methylation/deamination process and other genetic factors (e.g., recombination) influenced sequence composition changes and CGI evolution in vertebrate genomes.

We applied three different algorithms to identify CGIs in the fish genome. The TJ and GF algorithms have been frequently applied to scan CGIs in mammalian genomes and the CpGcluster was developed recently. The three algorithms gave out a much different number of CGIs, CGI density, and CGI length distribution, but our evaluation clearly indicated that the criteria of GF and CpGcluster algorithms were too generous. For example, for many genes that had one CGI per gene locus identified by TJ’s algorithm, we often found more than one but shorter CGI scattered in the same region by GF’s algorithm or CpGcluster. Overall, our evaluation suggests that TJ’s algorithm is likely most suitable for CGI identifications in fish genomes. Because high quality gene annotations or high-throughput experimental verification of CGIs has not made available in nonmammalian genomes, an evaluation of gene-associated CGIs is restricted at present. Thus, although the conclusions in this study would hold by any of the three algorithms, caution should be used when identifying CGI(s) for a specific gene in a nonmammalian genome, especially in a cold-blooded vertebrate genome.

## Figures and Tables

**Table 1:** Summary of five fish genomes. CpG islands in this table were identified by Takai and Jones’ algorithm [7]. The number of genes in each fish genome was based on the Ensembl database (http://www.ensembl.org/, build 49). Obs_CpG_/Exp_CpG_: the ratio of the observed over the expected CpG dinucleotides in a fish genome.

Common name	Species name	Length (Mb)	Number of genes	GC content (%)	Obs_CpG_/Exp_CpG_	Number of CGIs	CGI density (/Mb)
Tetraodon	*Tetraodon nigroviridis*	187	28 639	45.9	0.601	30 175	161.6
Stickleback	*Gasterosteus aculeatus*	391	22 310	44.5	0.662	61 768	157.8
Medaka	*Oryzias latipes*	582	20 159	40.1	0.479	21 522	37.0
Zebrafish	*Danio rerio*	1524	25 582	36.5	0.531	22 392	14.7
Fugu	*Takifugu rubripes*	351	19 244	45.5	0.565	47 251	134.5
